# FGF5 Regulates Schwann Cell Migration and Adhesion

**DOI:** 10.3389/fncel.2020.00237

**Published:** 2020-08-04

**Authors:** Bing Chen, Rong Hu, Qing Min, Yankun Li, David B. Parkinson, Xin-peng Dun

**Affiliations:** ^1^Department of Neurology, The Affiliated Huai’an No.1 People’s Hospital of Nanjing Medical University, Huai’an, China; ^2^School of Traditional Chinese Medicine, Southern Medical School, Guangzhou, China; ^3^School of Pharmacy, Hubei University of Science and Technology, Xianning, China; ^4^Peninsula Medical School, Faculty of Health, University of Plymouth, Plymouth, United Kingdom; ^5^The Co-innovation Center of Neuroregeneration, Nantong University, Nantong, China

**Keywords:** FGF5, receptors, peripheral nerve injury, Schwann cell, migration, adhesion, N-cadherin

## Abstract

The fibroblast growth factor (FGF) family polypeptides play key roles in promoting tissue regeneration and repair. FGF5 is strongly up-regulated in Schwann cells of the peripheral nervous system following injury; however, a role for FGF5 in peripheral nerve regeneration has not been shown up to now. In this report, we examined the expression of FGF5 and its receptors FGFR1-4 in Schwann cells of the mouse sciatic nerve following injury, and then measured the effects of FGF5 treatment upon cultured primary rat Schwann cells. By microarray and mRNA sequencing data analysis, RT-PCR, qPCR, western blotting and immunostaining, we show that FGF5 is highly up-regulated in Schwann cells of the mouse distal sciatic nerve following injury, and FGFR1 and FGFR2 are highly expressed in Schwann cells of the peripheral nerve both before and following injury. Using cultured primary rat Schwann cells, we show that FGF5 inhibits ERK1/2 MAP kinase activity but promotes rapid Schwann cell migration and adhesion *via* the upregulation of N-cadherin. Thus, FGF5 is an autocrine regulator of Schwann cells to regulate Schwann cell migration and adhesion.

## Introduction

The fibroblast growth factor (FGF) family includes 22 structurally related polypeptides that are highly conserved in mammals (Beenken and Mohammadi, 2009; Ornitz and Itoh, 2015). FGF1-22 proteins bind with high affinity to four FGF receptors, FGFR1-4, and FGFR1-4 activation has been implicated in multiple biological processes in mammals including cell proliferation and differentiation during development and tissue repair (Ornitz and Itoh, 2015). Mutations in FGF or FGFR genes cause developmental and genetic diseases in many different tissue types (Beenken and Mohammadi, 2009; Ornitz and Itoh, 2015). Numerous studies have also reported roles for FGFs in tissue regeneration and repair, underlying the importance of FGF-FGFR signaling in tissue homeostasis (Nunes et al., 2016; Charoenlarp et al., 2017; Maddaluno et al., 2017). The activation of a specific FGF-FGFR signaling and subsequent biological activities depends on the spatial and temporal expression pattern of each FGF ligand and their receptors (Beenken and Mohammadi, 2009; Ornitz and Itoh, 2015). We are interested in the molecular mechanism of peripheral nerve regeneration following injury, and among all the FGFs, FGF5 is highly up-regulated in the distal nerve in response to injury (Moscoso et al., 1998; McGeachie et al., 2001; Scarlato et al., 2001); this striking increase suggests a role for FGF5 in the regulation of peripheral nerve repair.

The FGF5 gene was first isolated as a human oncogene by a DNA rearrangement accompanying the transfection of fibroblasts with human tumor DNA (Zhan et al., 1987, 1988). FGFs can be arranged into seven subfamilies based on a phylogenetic analysis, and FGF5 resides in the FGF4 family, which is comprised of FGF4, FGF5, and FGF6 (Ornitz and Itoh, 2015). All members of this subfamily are secreted proteins with cleavable N-terminal signal peptides that mediate biological responses as extracellular proteins by binding to and activating FGFR1-4. Despite high expression of FGF5 in both embryonic and adult tissues (Haub et al., 1990; Haub and Goldfarb, 1991), FGF5 knockout mice are viable (Hébert et al., 1994) and the only phenotype that has been observed in FGF5 knockout mice was abnormally long hair (Hébert et al., 1994). This phenotype is identical to homozygous mice for the spontaneous mutation angora (*go*) mice (Hébert et al., 1994). Crossing of FGF5 knockout mice and go mutation mice failed to complement the long hair phenotype, resulting in the identification of the go mutant as a mutant allele of FGF5 (Hébert et al., 1994).

FGF5 is detectable in adult rat skeletal muscle and its expression increases in skeletal muscle following denervation (Hughes et al., 1993; McGeachie et al., 2001). *In vitro* analysis showed that FGF5 could promote the survival of embryonic motor neurons, therefore, FGF5 has been proposed as a muscle-derived regulator of motor axon regeneration (Hughes et al., 1993). However, *in vivo* studies have failed to show defects in muscle reinnervation in FGF5 null mice (Moscoso et al., 1998). Furthermore, *in vivo* studies in *go* homozygotes mice not only found that endogenous FGF5 is not transported in motor axons but also failed to reveal any loss of motoneurones (McGeachie et al., 2001). Later studies confirmed that FGF5 protein was expressed in the terminal and non-terminal Schwann cells but not in muscle fibers (McGeachie et al., 2001). Scarlato et al. (2001) also showed that nerve injury resulted in an increase of FGF5 in the Schwann cells of the distal nerve. Schwann cells have been shown to express FGFR1–3 (Meisinger and Grothe, 1997; Grothe et al., 2006; Furusho et al., 2009). This raised the possibility that FGF5 could be an autocrine regulator of Schwann cell behavior during nerve regeneration (McGeachie et al., 2001; Scarlato et al., 2001), however, the effects of FGF5 upon Schwann cells have not been examined.

In this report, we first systematically examined the expression of FGF5 and FGFR1-4 expression in Schwann cells upon injury, and then tested the effects of FGF5 on cultured primary rat Schwann cells. We show that FGF5 ligand is strongly up-regulated in mouse Schwann cells following injury, and FGFR1 and FGFR2 are highly expressed in Schwann cells of the mouse distal sciatic nerve. Using cultured primary rat Schwann cells, we show that FGF5 treatment rapidly promotes Schwann cell migration and adhesion *via* the upregulation of N-cadherin, identifying an autocrine function for FGF5 upon Schwann cells that regulates Schwann cell migration and adhesion.

## Materials and Methods

### Animals and Peripheral Nerve Surgery

All work involving animals was carried out according to Home Office regulation under the UK Animals Scientific Procedures Act 1986. Ethical approval for all experiments was granted by Plymouth University Animal Welfare and Ethical Review Board. Sprague–Dawley rats and C57BL/6 mouse breeding pairs were purchased from Charles River UK limited. PLP-GFP mice were described before Mallon et al. (2002) and Dun et al. (2019). All animals were housed in a controlled laboratory environment (temperature 22 ± 2°C, humidity 50–60%, 12-h light/dark cycle). All animals were fed with standard rodent diet and water added *ad libitum*. Two-month-old male and female mice were randomized and anesthetized with isoflurane, the right sciatic nerve was exposed and transected at approximately 0.5 cm proximal to the nerve trifurcation site and no re-anastomosis of the severed nerve was performed. The overlying muscle was sutured and the skin was closed with an Autoclip applier. All animals undergoing surgery were given appropriate post-operative analgesia, 0.05% bupivacaine solution, topically applied above the muscle suture before applying surgical clips. Meloxicam (5 mg/kg) injection was given just before recovery from anesthetic. All animals undergoing surgery were given nesting material and cage enrichment to minimize the risk of autotomy and monitored daily. At the indicated time points post-surgery for each experiment described, animals were euthanized humanely using carbon dioxide following UK Home Office regulations.

### FGF5, Primary and Secondary Antibodies

Recombinant Human FGF5 (R&D, 237-F5), anti-FGF5 antibody (Abcam, ab88118), anti-FGFR1 antibody (Abcam, ab10646), anti-FGFR2 antibody (Abcam, ab10648), CD206 antibody (R&D, AF2535), Phospho-ERK1/2 antibody (Cell Signaling, #9101), Total-ERK1/2 antibody (Cell Signaling, #9102), N-cadherin (Becton-Dickinson, 610920) and GAPDH antibody (EMD Millipore, MAB374) were used. Hoechst and species-specific secondary antibodies conjugated with Alexa Fluor 488 or 568 dyes were purchased from Invitrogen. Horseradish peroxidase (HRP) conjugated secondary antibodies for western blotting were purchased from Sigma.

### mRNA Purification, cDNA Synthesis, RT-PCR and qRT-PCR

Total mRNA was extracted using a miRNeasy Mini Kit (Qiagen, 217004) and the first-strand cDNA was synthesized with M-MLV reverse transcriptase (Promega, M368) using random hexamer primers (Promega, C1181). RT-PCR was performed in the G-Storm GS4M, qRT-PCR was performed in the PCR LightCycler480 Real-Time PCR Instrument (Roche Applied Science) using SYBR Green I Master with the primers showing in Table 1. Crosspoint (Cp) values were calculated by using the software of the LightCycler480 Real-Time PCR Instrument. Relative mRNA levels were calculated by the 2(-Delta Delta C(T)) method (Livak and Schmittgen, 2001) using GAPDH as a reference gene for normalization. All reactions were carried out in triplicate for statistical analysis.

**Table 1:** Sequence information of forward and reverse primers.

Primers	Forward: 5′—3′	Reverse: 5′—3′	Size
FGF5	CCCACGAAGCCAGTGTGTTA	ATCGCGGACGCATAGGTATT	199 bp
FGFR1	GACTCTGGCCTCTACGCTTG	TGGGGATGTCCAGTAGGGAG	194 bp
FGFR2	CACGACCAAGAAGCCAGACT	CTCGGCCGAAACTGTTACCT	94 bp
FGFR3	GTGGTGGCAGCTGTGATACT	TTAAGCGGGAAGCGAGAGAC	95 bp
FGFR4	GGAAGGTGGTCAGTGGGAAGT	CTGCTCCAGGATTGGGGCTA	164 bp
GAPDH	AAGGTCATCCCAGAGCTGAA	CTGCTTCACCACCTTCTTGA	222 bp

### Schwann Cell Culture and FGF5 Treatment

Schwann cells were prepared from the sciatic nerve and brachial plexus of day three Sprague–Dawley rats as previously described (Brockes et al., 1979; Dong et al., 1997; Maurel, 2018). Schwann cells were cultured in six well plates with low glucose (1 g/ml) DMEM containing 10% fetal bovine serum (FBS), 10 ng/ml NRG-1 (R&D, Cat No. 396-HB-050) and 2 μM forskolin (Sigma, Cat No. 344270). For the ERK activation experiment, Schwann cells were starved in low glucose DMEM containing 1% FBS overnight. The following morning cells were treated with 10 ng/ml FGF5 and lysed after 0, 5, 10, 20, 30, and 60 min of treatment. For extended FGF5 treatment, Schwann cells were changed to low glucose DMEM containing 10% FBS 16 h before FGF5 treatment, and the following morning treated with 5 ng/ml FGF5 and images were taken every 2 h on a LeicaIM8 microscope.

### Immunohistochemistry and Immunocytochemistry

Sciatic nerve samples were dissected out and fixed overnight in 4% paraformaldehyde (in PBS, PH7.2) at 4°C. The samples were then washed in PBS (3 × 10 min) and dehydrated in 30% sucrose (in PBS) overnight at 4°C. Subsequently, samples were embedded in OCT medium and sectioned on a cryostat at a thickness of 12 μm. Schwann cells cultured on coverslips were fixed in 4% paraformaldehyde at 4°C for 15 min and then washed in PBS (3 × 10 min). The sections or cells were permeabilized with 0.25% Triton X-100 plus 1% bovine serum albumin (BSA) in PBS for 45 min and then blocked with blocking buffer (3% BSA plus 0.05% Triton X-100 in PBS) for 1 h at room temperature. The sections or cells were incubated with primary antibodies (1:100 diluted in blocking buffer) overnight at 4°C. The next day, sections were washed with PBS (3 × 10 min) and then incubated with species-specific secondary antibodies plus Hoechst dye (1:500 diluted in blocking buffer) for 1 h at room temperature. Finally, sections were washed with PBS (3 × 10 min) and mounted with Citifluor (Agar Scientific, R1320) for imaging with a Leica SPE confocal microscope (Dun et al., 2019).

### Western Blot

Nerve samples were dissected out with similar size and directly sonicated into 1× SDS loading buffer. Cells were lysed in 200 μl of radio-immunoprecipitation assay (RIPA) buffer (50 mM Tris-HCl, pH 7.4, 0.1% SDS, 1% NP-40, 150 mM NaCl, 1 mM ethylenediaminetetraacetic acid (EDTA), 0.5% sodium deoxycholate) plus phosphatase inhibitor cocktails (1:100, Santa Cruz Biotechnology, sc-45045 and sc-45065). Cell lysates were spun down at 16,000× *g* for 15 min at 4°C. The supernatant was transferred to new 1.5 ml microcentrifuge tubes and the protein concentration was determined using the Pierce™ BCA Protein Assay Kit. An appropriate volume of samples containing 20 μg of protein was added to the 4× sample buffer. Proteins were separated on 10% or 12% SDS polyacrylamide running gels and transferred onto a polyvinylidene fluoride (PVDF, 0.45 μm) transfer membrane using the wet transfer method. Membranes were blocked in 5% fat-free milk in TBST (Tris-buffered saline plus 0.1% Tween-20) for 1 h at room temperature. Primary antibodies were diluted (1:500) in 5% milk (in TBST) and the membranes were incubated in primary antibodies overnight at 4°C. The next day, membranes were washed in TBST (3 × 10 min) and then incubated with HRP conjugated secondary antibody (Sigma, 1:5,000 in 5% milk, TBST) for 1 h at room temperature. After three TBST washes (10 min each), Pierce ECL western blotting substrate was added onto the membrane and incubated for 5 min to develop the chemiluminescent signal. Amersham Hyperfilm™ ECL films were used to capture the intensity of the chemiluminescent signal. Exposed films were then developed in a Compact X4 automatic processor. The intensity of protein bands was quantified using the free ImageJ software available from https://imagej.nih.gov/ij/.

### Cell Viability Assay

Culturing medium in the 6-well plate was carefully removed and Schwann cells were washed once with 1 ml PBS. Schwann cells were then stained with a 1.5 ml trypan blue solution (0.4%, Thermo Fisher Scientific, Cat No.:15250061; Strober, 2015; Lebeau et al., 2019). After 2 min staining, cells were rinsed twice with 1 ml PBS and a final 1.5 ml PBS was added into the wells for immediately imaging using a LeicaIM8 microscope.

### Statistical Analysis

The samples for Western blotting and qRT-PCR were prepared by grouping three nerves from three different mice together for each time point to create a pooled sample as *n* = 1, and then repeated the process using another six animals to reach *n* = 3. Therefore, we have used pooled biological replicates for the repetition of these experiments. Statistical significance was analyzed using the Student’s *t*-test by comparing the test groups with the control groups. Data are represented in the figures as mean value ± SEM.

## Results

### Analysis of FGF5 Up-Regulation in Schwann Cells and in the Mouse Distal Sciatic Nerve With Published Microarray and mRNA Sequencing Data Sets

**Figure 1:**
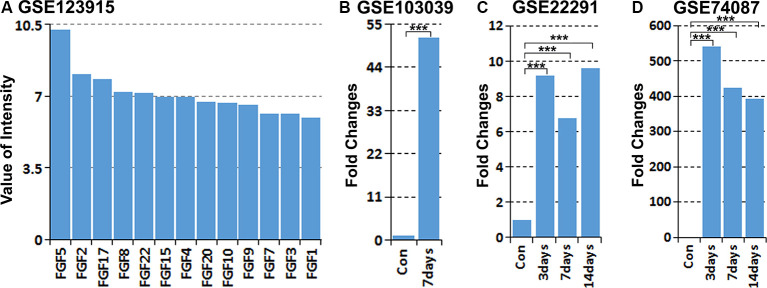
Analysis of fibroblast growth factor 5 (FGF5) up-regulation in Schwann cells and in the mouse distal sciatic nerve from published microarray and mRNA sequencing data sets. **(A)** Analyzing the expression of all FGFs in cultured rat primary Schwann cells with our microarray data GSE123915, FGF5 has the highest expression level in cultured rat primary Schwann cells. **(B)** Analyzing FGF5 mRNA up-regulation in Schwann cells of the distal mouse sciatic nerve 7 days post-injury with the mRNA sequencing data set GSE103039, FGF5 mRNA level is 51.5-fold upregulated in Schwann cells compared to the uninjured nerve. **(C,D)** Analyzing microarray data sets GSE22291 and GSE74087 showing FGF5 mRNA up-regulation in the mouse distal sciatic nerve at 3, 7, and 14 days post-injury. ***Indicates *p* < 0.001.

Previously, we studied the gene expression profile of cultured rat primary Schwann cells by microarray analysis (Dun et al., 2019). Analyzing the expression of all FGF family members in our microarray data (GSE123915) showed that FGF5 has the highest expression level in cultured rat primary Schwann cells (Figure 1A). Recently, Clements et al. (2017) studied *in vivo* Schwann cell mRNA expression by an mRNA sequencing technique with Schwann cells isolated from intact and 7 days injured mouse sciatic nerve (GSE103039). Analyzing their mRNA sequencing data showed that FGF5 mRNA is up-regulated greater than 50-fold in Schwann cells following injury (Figure 1B). We further analyzed two published microarray data sets, GSE22291 and GSE74087, in the Gene Expression Omnibus (GEO^1^) that have studied the time course of gene expression in adult mouse distal sciatic nerve at 3, 7 and 14 days post-injury (Barrette et al., 2010; Pan et al., 2016). Although FGF5 fold changes have large differences between two data sets of GSE22291 and GSE74087, analyzing both data sets showed that FGF5 is highly up-regulated in the mouse distal sciatic nerve at 3, 7 and 14 days following injury (Figures 1C,D).

### Validation of FGF5 Increase in Schwann Cells of the Mouse Distal Sciatic Nerve Following Injury

**Figure 2:**
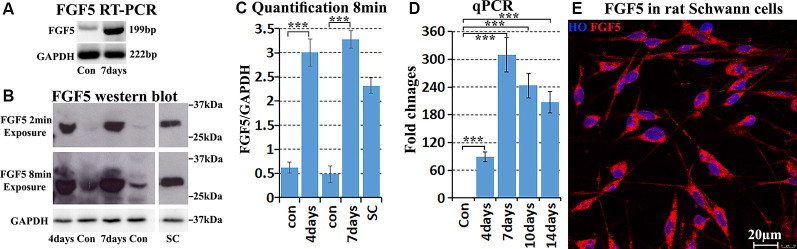
Validation of FGF5 up-regulation in mouse distal sciatic nerve and cultured Schwann cells. **(A)** RT-PCR showing weak FGF5 mRNA expression in control sciatic nerve and strong expression in the distal sciatic nerve at 7 days post-injury. **(B)** Western blot detecting weak FGF5 protein expression in control mouse sciatic nerve and strong up-regulation in distal sciatic nerve at 4 and 7 days post-injury. Cultured rat primary Schwann cells express a high level of FGF5 protein. **(C)** Quantification of three independent FGF5 western blot results. **(D)** Quantitative PCR validates FGF5 mRNA up-regulation in the mouse distal sciatic nerve at 4, 7, 10, and 14 days post-injury; intact contralateral sciatic nerves used as control samples. **(E)** Staining of FGF5 on cultured rat primary Schwann cells shows punctate FGF5 expression pattern in Schwann cell cytoplasm. HO: Hoechst. Scale bar in **(E)** 20 μm. ***Indicates *p* < 0.001.

Next, we validated FGF5 mRNA up-regulation in mouse distal sciatic nerve following injury. RT-PCR results showed weak FGF5 expression on intact adult mouse sciatic nerve with an upregulation in the distal sciatic nerve at 7 days following injury (Figure 2A). Quantitative PCR showed that FGF5 was significantly up-regulated from day 4 to day 14 in the mouse distal sciatic nerve compared to intact control contralateral nerve (Figure 2D). On day 4 after injury, FGF5 mRNA showed an almost 90-fold up-regulation compared to uninjured controls, with an even higher increase seen (>300) at 7 days and with a greatly increased expression at days 10 and 14 (Figure 2D). The FGF5 mRNA fold changes from our qPCR data show high similarity with the microarray data set GSE74087 (Figure 1D). Consistent with the results of the FGF5 mRNA expression pattern, FGF5 protein measurement by Western blotting is low in the intact mouse sciatic nerve and is highly up-regulated in the distal sciatic nerve following injury (Figures 2B,C). Our western blot results also showed that cultured rat primary Schwann cells express high levels of FGF5 protein (Figures 2B,C). Staining FGF5 on cultured rat primary Schwann cells revealed that FGF5 shows a punctate expression pattern in Schwann cell cytoplasm (Figure 2E), a typical staining pattern for secreted polypeptides (van Lieshout et al., 1995; Whim and Moss, 2001).

**Figure 3:**
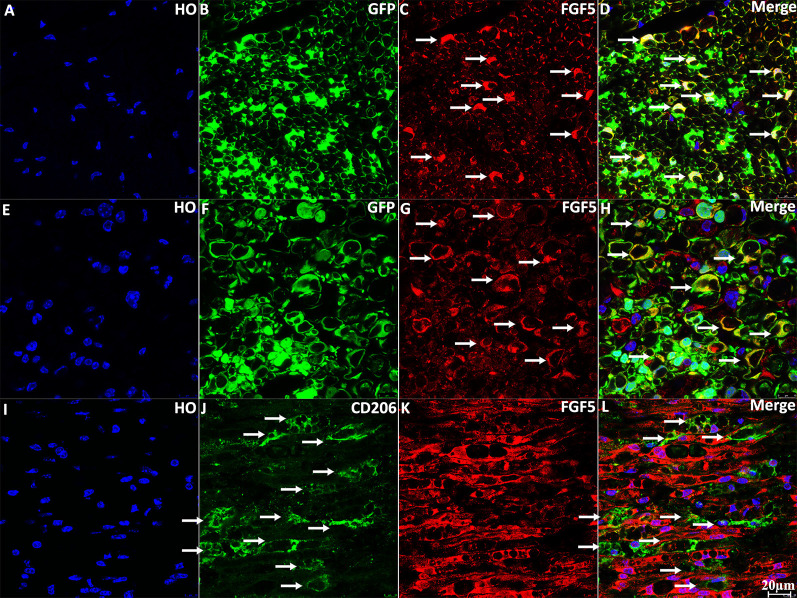
Staining of FGF5 in intact and 7 days post-injury mouse sciatic nerve. **(A–D)** Staining of FGF5 on intact sciatic nerve transverse sections from PLP-GFP mice. FGF5 can be seen in the cell bodies of myelinating Schwann cells (indicated by arrows in panel **C**). **(E–H)** Staining of FGF5 on distal sciatic nerve transverse sections from PLP-GFP mice at 7 days post-injury; Schwann cells are the principal cells expressing FGF5 in the distal sciatic nerve. **(I–L)** Double staining of FGF5 with the macrophage marker CD206 on longitudinal sections of the distal sciatic nerve from C57BL/6 mice at 7 days post-injury; FGF5 is not expressed in macrophages of the distal sciatic nerve. HO: Hoechst. Scale bar 20 μm.

To confirm the expression of FGF5 in Schwann cells of the sciatic nerve, we stained FGF5 on intact and injured sciatic nerve sections from PLP-GFP mice, which label Schwann cells GFP-positive (Mallon et al., 2002; Carr et al., 2017; Dun et al., 2019). Staining of FGF5 on intact sciatic nerve transverse sections from PLP-GFP mice showed that FGF5 could be easily detected in the cell bodies of myelinating Schwann cells (Figures 3A–D, indicated by white arrows). Staining for FGF5 on injured nerves of PLP-GFP mice at 7 days post-injury revealed that Schwann cells are the principal cells expressing FGF5 (Figures 3E–H, indicated by white arrows). Previously, McGeachie et al. (2001) found that macrophages in skeletal muscle also express FGF5 after denervation. Resident and infiltrated macrophages are an important cell type in the distal nerve promoting peripheral nerve regeneration following injury and comprise 20% of cells in the distal sciatic nerve (Stierli et al., 2018). Ydens et al. (2012) showed that the majority of macrophages in the distal nerve show an M2 phenotype. Therefore, we double stained FGF5 with an M2 macrophage marker CD206 on distal sciatic nerve longitudinal sections from C57BL/6 mice at 7 days post-injury. Our staining showed that FGF5 is not expressed in M2 macrophages of the distal sciatic nerve (Figures 3I–L, indicated by white arrows).

### FGF5 Receptors Expression in Schwann Cells of the Mouse Distal Sciatic Nerve Following Injury

**Figure 4:**
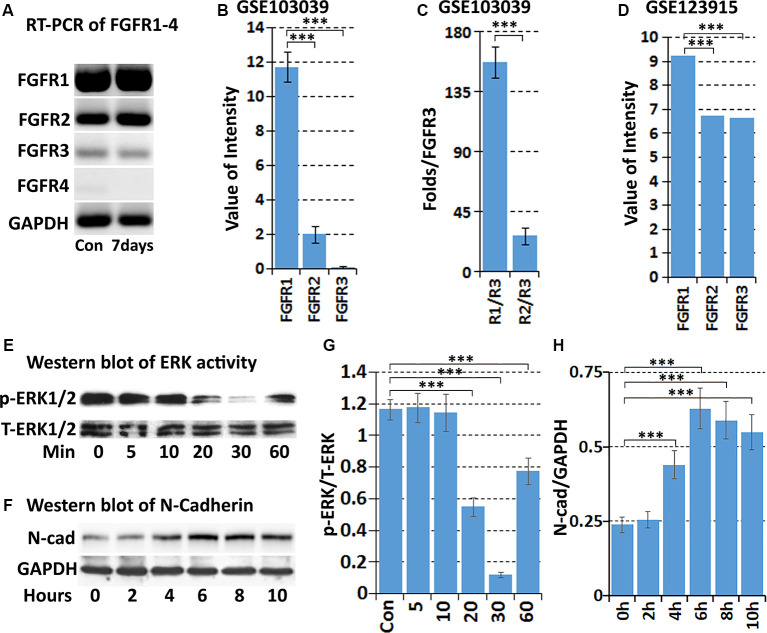
Expression of FGFR1-4 in intact and injured mouse sciatic nerve. **(A)** RT-PCR showing FGFR1-4 mRNA expression in control and 7 days post-injury mouse sciatic nerve. **(B)** Data showing expression of FGFR1–3 mRNA at 7 days post-injury from mRNA sequencing data set GSE103039. **(C)** Fold difference of FGFR1 and FGFR2 against FGFR3 analyzed with the mRNA sequencing data set GSE103039. **(D)** The expression value of FGFR1–3 in cultured Schwann cells in our microarray data set GSE123915. **(E)** Western blot results showing that FGF5 inhibits the basal levels of ERK1/2 activity in cultured rat primary Schwann cells. **(F)** Western blot results showing that FGF5 significantly upregulates N-cadherin expression in Schwann cells after 4, 6, 8, and 10 h treatment. **(G)** Quantification of three independent western blot results for ERK1/2 inhibition by FGF5. **(H)** Quantification of three independent western blot results for N-cadherin upregulation. ***Indicates *p* < 0.001.

The FGF family molecules can bind to FGFR1-4 and regulate migration, proliferation, differentiation, survival, and metabolic activities in a wide variety of cells (Ornitz and Itoh, 2015). To understand the role of FGF5 in Schwann cells during peripheral nerve regeneration, we used RT-PCR to detect FGFR1-4 expression in intact mouse sciatic nerve, and in the distal sciatic nerve following injury. Results showed that FGFR1–3 mRNAs are present in the intact and injured mouse sciatic nerve but FGFR4 is hardly detectable both before and after injury (Figure 4A). FGFR1 appears at the highest level of expression in the mouse sciatic nerve both before and after injury. FGFR2 is also expressed, but the expression of FGFR3 is very weak (Figure 4A). We next analyzed FGFR1-4 mRNA expression in Schwann cells isolated from intact and 7 days injured mouse sciatic nerve with the data set of GSE103039 (Clements et al., 2017). Consistent with our RT-PCR results, FGFR1–3 mRNA could be detected in isolated Schwann cells by mRNA sequencing both before and after injury but FGFR4 mRNA was undetectable (Clements et al., 2017). Analyzing the mRNA sequencing data of the Clements dataset also showed that Schwann cells in the mouse distal sciatic nerve express the highest levels of FGFR1, followed by FGFR2 and then FGFR3 (Figures 4B,C). We also analyzed FGFR1–3 expression on cultured rat primary Schwann cells with our microarray data set GSE123915 (Dun et al., 2019). In agreement with the mRNA sequencing data of FGFR1–3 expression levels *in vivo* for Schwann cells, relative expression levels of FGFR1>FGFR2>FGFR3 were observed (Figure 4D).

**Figure 5:**
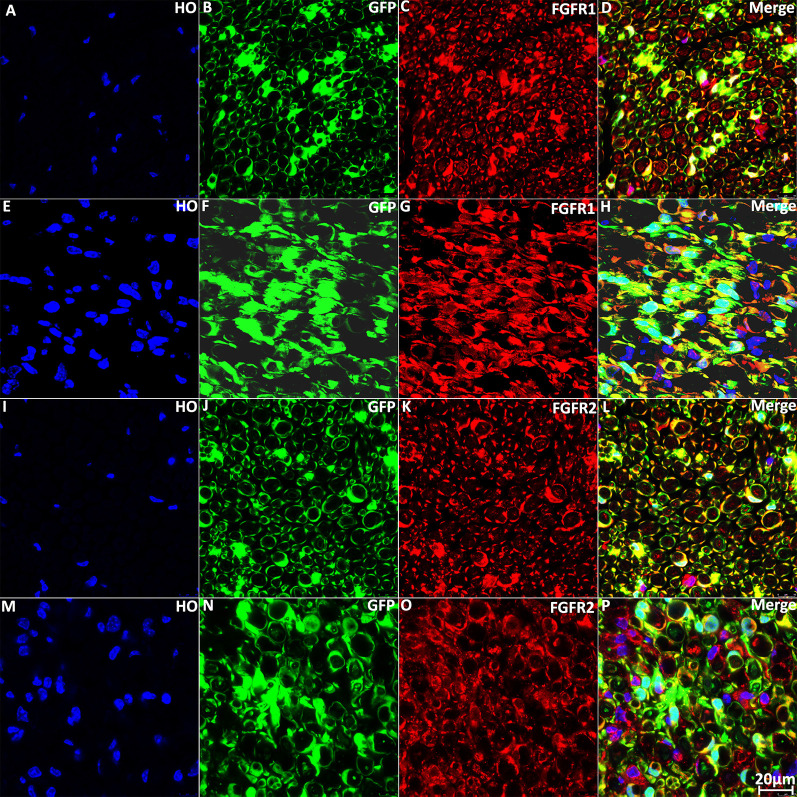
Staining of FGFR1 and FGFR2 in intact and injured mouse sciatic nerve of PLP-GFP mice. **(A–D)** Staining of FGFR1 on intact sciatic nerve transverse sections from PLP-GFP mice. **(E–H)** Staining of FGFR1 on distal sciatic nerve transverse sections from PLP-GFP mice at 7 days post-injury; Schwann cells are the principal cells expressing FGFR1 in the distal sciatic nerve. **(I–L)** Staining of FGFR2 on intact sciatic nerve transverse sections from PLP-GFP mice. **(M–P)** Staining of FGFR2 on distal sciatic nerve transverse sections from PLP-GFP mice at 7 days post-injury, Schwann cells in the distal sciatic nerve express FGFR2. HO: Hoechst. Scale bar 20 μm.

Next, we immunolabeled for FGFR1 and FGFR2 on intact and injured sciatic nerve sections from PLP-GFP mice. Staining for FGFR1 and FGFR2 on intact sciatic nerve transverse sections showed that FGFR1 and FGFR2 are both primarily expressed on Schwann cells of the sciatic nerve (Figure 5). Staining of FGFR1 and FGFR2 on distal sciatic nerve transverse sections from PLP-GFP mice at 7 days post-injury revealed that Schwann cells are the principal cells expressing FGFR1 (Figures 5E–H) and FGFR2 (Figures 5M–P) in the distal sciatic nerve.

### FGF5 Regulates Schwann Cell Migration and Adhesion

Initially, FGF5 was suggested to have a function in promoting the survival of motoneurons (Hughes et al., 1993), although a loss of motoneurons in go mutation homozygous mice was not observed (McGeachie et al., 2001). A regeneration defect of motor axon reinnervation was also not observed in go mutation homozygous mice (Moscoso et al., 1998). Based on the expression of FGFR1 and FGFR2 on Schwann cells, FGF5 has been suggested as an autocrine regulator for Schwann cell proliferation and survival during Wallerian degeneration (McGeachie et al., 2001; Scarlato et al., 2001), but this possible FGF5 function remains to be examined. FGF-mediated cell proliferation and survival are primarily triggered by the activation of downstream mitogen-activated protein kinase (MAPK) signaling pathways (Ornitz and Itoh, 2015; Tian et al., 2019). Therefore, we treated cultured rat primary Schwann cells with FGF5 and examined ERK1/2 activation by western blot. To our surprise, FGF5 treatment failed to activate ERK1/2 in Schwann cells but instead inhibited the basal level of ERK1/2 activity (Figures 4E,G).

**Figure 6:**
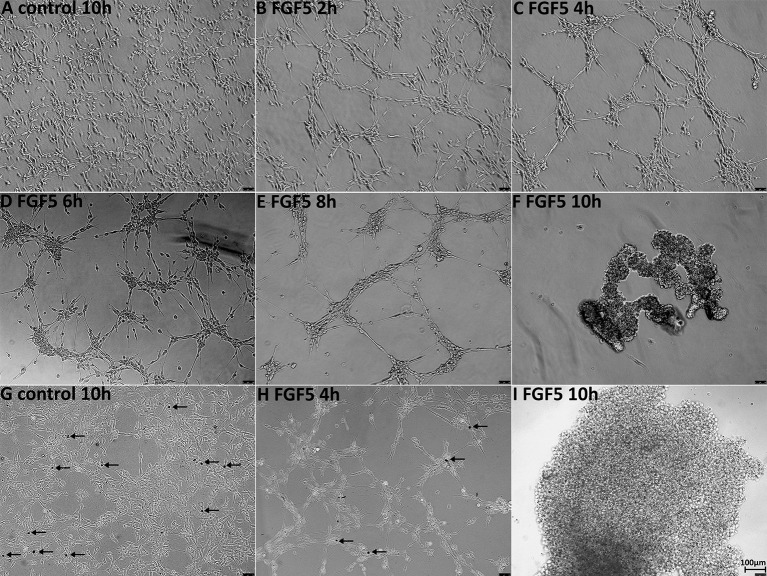
FGF5 regulates Schwann cell migration and adhesion. **(A)** Phase-contrast image of control untreated rat primary Schwann cells. **(B–F)** Phase-contrast images of FGF5-treated (5 ng/ml) rat primary Schwann cells at timepoints 2, 4, 6, 8, and 10 h. **(G–I)** Phase-contrast images show cell viability in control and FGF5-treated Schwann cells, stained by trypan blue. Arrows in panels **(G,H)** indicate dead cells. At 10 h of FGF5 treatment in panel **(I)**, there were no dead cells stained by trypan blue in Schwann cell clusters. Scale bar 100 μm.

Having not observed ERK1/2 activation in Schwann cells up to 1 h following FGF5 treatment, we extended the time of FGF5 treatment to examine FGF5 effects on Schwann cells. Interestingly, we observed rapid Schwann cell movement after 2 h of treatment and increased cell-cell adherence (Figure 6B). Following FGF5 treatment, Schwann cells could be observed moving toward each other and forming distinct clusters, in a time-dependent manner with Schwann cell processes connecting adjacent clusters (Figure 6). The Schwann cell cluster formation indicated that FGF5 not only regulates rapid Schwann cell movement but also strongly enhances Schwann cell adhesion. At 6 h of treatment, lager Schwann cell clusters could be observed and more larger space appeared in the wells of six-well plates, Schwann cells within the same cluster could be observed growing on top of each other. At this stage, some individual Schwann cells could be seen but they are sending a long process connecting with surrounding clusters (Figure 6D). By the time of 8 h of treatment, small clusters have moved toward each other and formed even larger clusters. The Schwann cell processes connecting different clusters still could be seen, but it is rare to see individual Schwann cells in space (Figure 6E). It appears that the connecting processes could communicate between cells and promote adjacent clusters moving together to form larger clusters. At 10 h of treatment, all the clusters moved together and formed three or four big clusters in each well of the six-well plate (Figure 6F). We further performed trypan blue exclusion staining to test the cell viability because most cell clusters detached from the wells after 10 h of FGF5 treatment. The staining showed that Schwann cells are alive although they formed clusters and detached from the wells (Figures 6G–I).

Previous studies showed that N-cadherin is upregulated in Schwann cells following peripheral nerve injury and N-cadherin is a key adhesion molecule that regulates Schwann cell migration and adhesion (Wanner and Wood, 2002; Parrinello et al., 2010; Clements et al., 2017; Roberts et al., 2017). We then examined N-cadherin expression in Schwann cells by western blot following FGF5 treatment. The western blot results revealed that N-cadherin expression is significantly upregulated in Schwann cells after 4, 6, 8, and 10 h of FGF5 treatment (Figures 4F,H). Thus, we identified an FGF5 autocrine function regulating Schwann cell migration and adhesion *via* the upregulation of N-cadherin.

## Discussion

The FGF family consists of 22 structurally related molecules that bind with high affinity to four FGF receptors (FGFR1-4). Activation of FGF-FGFR signaling and subsequent biological activities not only depend on the spatial and temporal expression pattern of FGFs but also the expression patterns of the FGFR1-4 receptors (Ornitz and Itoh, 2015; Tian et al., 2019). Numerous recent studies have reported roles for the FGFs in tissue and organ regeneration, such as FGF2, FGF4, and FGF8 for the limb/digit regeneration, FGF1 and FGF8 for lens regeneration, FGF2 for nervous system regeneration and FGF7 for skin and intestinal regeneration (Nunes et al., 2016; Maddaluno et al., 2017). Among all the FGFs, FGF1 and FGF2 have been studied in peripheral nerve regeneration. FGF1 expression is restricted largely to neurons, therefore levels of FGF1 protein fall in the distal nerve following injury and axon degradation (Eckenstein et al., 1991; Ishikawa et al., 1992). FGF-2, in contrast, is expressed by Schwann cells both before and after peripheral nerve injury (Meisinger and Grothe, 1997; Grothe et al., 2006; Furusho et al., 2009). Overexpression of FGF2 promoted peripheral nerve regeneration and increased Schwann cell proliferation, axonal regrowth, and re-myelination (Jungnickel et al., 2006). Additionally, repairing of a nerve gap following transection with a silicone tube containing FGF2 overexpressing Schwann cells also promoted peripheral nerve regeneration (Allodi et al., 2014).

So far, the best-established biological effect of FGF5 is as a paracrine inhibitor of proliferation of hair outer root sheath cells (Pethö-Schramm et al., 1996; Higgins et al., 2014). In humans, FGF5 is expressed within the upper outer root sheath cells of hair follicles, with FGF5 mutation resulting in excessively long eyelashes (Higgins et al., 2014). Global FGF5 knockout mice are viable and have an abnormally long hair phenotype, which is also seen in many other mammalian species (Hébert et al., 1994). This phenotype is identical to that of mice homozygous for the spontaneous mutation angora (*go*) mice. FGF5 knockout mice and *go* mutations cross-breeding failed to rescue each other’s long hair phenotype, resulting in the identification of exon 1 deletion of FGF5 DNA in *go* mutant mice. Thus, *go* is a mutant allele of FGF5 (Hébert et al., 1994). The long hair phenotype has also been reported in other species. Genome-wide association studies in dogs identified a mutation in FGF5 that is associated with hair length (Housley and Venta, 2006; Dierks et al., 2013). A missense mutation in FGF5 was found in longhaired cats (Drögemüller et al., 2007; Kehler et al., 2007). The long-hair phenotype of FGF5 gene knockout or mutations has also been reported in sheep, goat, donkey, hamster and guinea pigs (Legrand et al., 2014; Wang et al., 2015, 2016; Yoshizawa et al., 2015, 2016; Hu et al., 2017; Li et al., 2017; Yu et al., 2018). These findings provide evidence that FGF5 functions as an inhibitor of hair elongation, thus identifying one of the FGF5 key functions.

Northern blot analysis of total RNA obtained from hindlimb skeletal muscle of embryonic, postnatal and adult rats showed that FGF5 mRNA is expressed in rat skeletal muscle during the period of embryonic motoneuron death as well as in the adult skeletal muscle (Hughes et al., 1993). Therefore, the potential role of FGF5 functioning as a target-derived trophic factor for spinal motoneurons has been examined *in vitro*. *In vitro* experiments showed that recombinant FGF5 promoted the survival of cultured embryonic chick motoneurons. Extracts from rat skeletal muscle also promoted the survival of cultured embryonic chick motoneurons and the motoneuron survival activity of rat skeletal muscle extracts could be immunoprecipitated using an antiserum to FGF5 (Hughes et al., 1993). Lindholm et al. (1994) also reported that FGF5 has neurotrophic activity on cultured rat septal cholinergic and raphe serotonergic neurons. Above *in vitro* results suggested that FGF5 might act as a target-derived trophic factor for motoneurons. However, *in vivo* studies using the double-ligation technique has revealed that endogenous FGF5 is not transported in motor axons. Furthermore, stereological estimates of the number of motoneurons in *go* homozygotes mice failed to reveal any loss of any motoneurons compared to control mice (McGeachie et al., 2001). Moreover, another study also failed to discover any abnormalities of the motor nerve terminal Schwann cells, synaptic basal lamina, or postsynaptic membrane in *go* homozygous mice (Moscoso et al., 1998). Taken together, these results demonstrated that FGF5 is dispensable for major aspects of the nervous system development.

While FGF5 is dispensable during nervous system development (Moscoso et al., 1998; McGeachie et al., 2001). McGeachie et al. (2001) showed that FGF5 mRNA was up-regulated 50-fold in denervated muscles at 7 days after denervation, and remained high for at least 28 days. They carefully examined the FGF5-positive cell types in rat skeletal muscles, showing that FGF5 mRNA and protein were expressed in both terminal and non-terminal Schwann cells of the skeletal muscles but not in muscle fibers (McGeachie et al., 2001). Scarlato et al. (2001) also reported that FGF5 is dramatically up-regulated in Schwann cells of the peripheral nerves following axotomy, but FGF5 levels gradually returned to normal upon the contact of regenerating axons with Schwann cells. Cultured Schwann cells express FGF5 and forskolin treatment elevates FGF5 expression in cultured Schwann cells. Their study provided evidence that axon-Schwann cell interaction regulates FGF5 levels in Schwann cell of the peripheral nerves as the time course of FGF5 level changes matches with the time course of axon degeneration and regeneration (Scarlato et al., 2001), although *in vivo* experiments in the go homozygous FGF5 mouse mutant failed to reveal any differences in the measured regeneration (Moscoso et al., 1998). Thus, an FGF5 function in peripheral nerve regeneration has not been identified with these studies.

Previously, Scarlato et al. (2001) reported that they could not detect FGF5 mRNA and protein in adult intact sciatic nerves. FGF5 is highly up-regulated following peripheral nerve injury, therefore, FGF5 has been named as one of the injury-induced genes (Ma et al., 2016). In contrast, McGeachie et al. (2001) demonstrated by RT-PCR that FGF5 mRNA is expressed in adult mouse sciatic nerve. In this study, we found that both FGF5 mRNA and protein could be detected in intact mouse sciatic nerve, and it is expressed by myelinating Schwann cells. Thus, FGF5 expression in the peripheral nerves is not purely induced by injury response.

The FGF-FGFR signaling pathways regulate important biological processes including cell proliferation and differentiation during development and tissue repair. Upon binding to their high-affinity receptors, FGFs activate various signaling cascades, of which the Ras-ERK1/2 signaling pathway is most prominent (Ornitz and Itoh, 2015). Recently, Tian et al. (2019) also reported that FGF5 promoted spermatogonial stem cell proliferation *via* ERK and AKT activation. Previous studies have suggested FGF5 as an autocrine regulator for Schwann cell proliferation and survival during Wallerian degeneration because Schwann cells in the distal nerve express FGFRs (Meisinger and Grothe, 1997; McGeachie et al., 2001; Scarlato et al., 2001; Grothe et al., 2006; Furusho et al., 2009). In this report, we showed that Schwann cells in the distal nerve express a high level of FGFR1 and FGFR2, and it has been reported that FGF5 could activate FGFR1 and FGFR2 with a Kd value of approximately 1 nM (Clements et al., 1993). This raised the possibility that FGF5 could be an autocrine regulator of Schwann cell proliferation following peripheral nerve injury. However, our FGF5 treatment inhibited the basal level of ERK1/2 activity in cultured rat primary Schwann cells, instead, it regulates Schwann cell migration and adhesion. In addition to the ERK signaling pathway, FGFs can activate the phosphatidylinositide 3-kinase/Akt, phospholipase Cγ, p38, and JNK kinases, STAT1, STAT3 and STAT5 pathways (Ornitz and Itoh, 2015). These pathways could be activated in a cell type-dependent manner (Ornitz and Itoh, 2015). Our results of FGF5 regulates Schwann cell migration and adhesion indicated that FGF5 could activate other signaling pathways in Schwann cells rather than ERK1/2 signaling. Our findings further support the evidence that FGFs could stimulate the proliferation of some cells but inhibit the proliferation and promote the differentiation of others.

In this report, we identified an FGF5 autocrine function regulating Schwann cell migration and adhesion *via* the upregulation of N-cadherin. Previous studies showed that N-cadherin is expressed in cultured rat primary Schwann cells (Wanner and Wood, 2002; Roberts et al., 2017), and N-cadherin is upregulated in Schwann cells following peripheral nerve injury (Parrinello et al., 2010; Clements et al., 2017; Roberts et al., 2017). Studies have shown that Schwann cells in the peripheral nerves promote the repair of multiple tissue types including peripheral nerve gap bridging, skin wound healing and digit tip regeneration (Johnston et al., 2016; Carr and Johnston, 2017; Parfejevs et al., 2018; Jones et al., 2019). The injury-induced changes not only reprogram denervated Schwann cells into a repair cell state but also give them a highly motile phenotype. The importance of migrating Schwann cells in tissue regeneration is most evident in the cases of peripheral nerve transection injuries (Parrinello et al., 2010; Cattin et al., 2015; Dun et al., 2019). Following peripheral nerve transection injury, Schwann cells from both nerve end migrate into the nerve gap and form Schwann cell cords in the nerve bridge to guide axons regeneration. This Schwann cell migration property is key to successful peripheral nerve regeneration (Parrinello et al., 2010; Cattin et al., 2015; Chen et al., 2019; Dun and Parkinson, 2020). During the period of migration in the nerve bridge, Schwann cells contact each other through their long processes and form a chain to migrate toward the middle of the nerve bridge (Chen et al., 2019). Schwann cell migration in the nerve bridge appears as a classic example of cell chain migration, and this chain migration is important for the correct Schwann cell cord formation in the nerve bridge (Aigouy et al., 2008; Parrinello et al., 2010; Dun and Parkinson, 2015). Parrinello et al. (2010) reported that N-cadherin is a key adhesion molecule in Schwann cells regulating chain formation in the nerve bridge during migration. Thus, our findings indicate that FGF5 could regulate N-cadherin expression in migrating Schwann cells in the nerve bridge and promote Schwann cell chain formation.

In summary, we first systematically examined the expression of FGF5 and FGFR1-4 expression in Schwann cells of the mouse distal sciatic nerve and then investigated the effect of FGF5 treatment upon cultured primary rat Schwann cells. By microarray and mRNA sequencing data set analysis, RT-PCR, qPCR, western blot and immunostaining studies, we show that FGF5 is highly up-regulated in Schwann cells of the mouse distal sciatic nerve, and its receptors FGFR1 and FGFR2 are highly expressed in Schwann cells of the peripheral nerve both before and after injury. Using cultured primary rat Schwann cells, we revealed that FGF5 inhibits ERK1/2 activation but FGF5 regulates rapid Schwann cell migration and adhesion. Thus, FGF5 is an autocrine regulator of Schwann cell migration and adhesion. So far, Schwann cells are the best cell type that could be delivered into a nerve guidance conduit to promote peripheral nerve regeneration. Currently, cell delivery requires the use of collagen or a matrix gel to support cells inside the nerve guidance conduit. The use of collagen or matrix gel often decreases cell density and prevent cell-cell interaction and communication. We believe this FGF5 property of regulating Schwann cell adhesion could be utilized for Schwann cell delivery to prepare better engineered neural tissue for peripheral nerve repair.

## Data Availability Statement

All datasets presented in this study are included in the article.

## Ethics Statement

The animal study was reviewed and approved by Plymouth University Animal Welfare and Ethical Review Board.

## Author Contributions

BC, RH, QM, and YL performed experiments and analyzed the data. XD designed the research. XD and DP wrote the article.

## Conflict of Interest

The authors declare that the research was conducted in the absence of any commercial or financial relationships that could be construed as a potential conflict of interest.

## Footnotes

^1^http://www.ncbi.nlm.nih.gov/geo/
